# Supplementary material for: Effects of Melatonin Alone or Associated with Acyclovir on the Suppressive Treatment of Recurrent Genital Herpes: A Prospective, Randomized, and Double-Blind Study
Source: Biomedicines. 2023 Apr 4;11(4):1088. doi: 10.3390/biomedicines11041088 (PMC10135732; doi:10.3390/biomedicines11041088)
Supplement: Supplementary file 1 [file biomedicines-11-01088-s001.zip › biomedicines-2219059-supplementary.pdf]

Supplementary material

Table S1 - Outpatient visits.

Selection	Before treatment	30 days	60 days	90 days	180 days	210 days (30 days after treatment)	240 days (60 days after treatment)
Screening	Presentation of the project<br>Consented form signature<br>Clinical history<br>Gynecological examination<br>Blood collection<br>Completing questionnaires<br>Medication delivery	Physical examination<br>Record adverse effects	Evaluation of correct medication intake<br>Record adverse effects	Evaluation of correct medication intake<br>Record adverse effects<br>Physical examination	Record adverse effects<br>Physical examination<br>Completing questionnaires<br>Blood collection	Test results and comparison with the onset of medication<br>Record adverse effects	Record of adverse effects<br>Revelation of the study arm

Table S2 – Exclusion criteria (treatment and follow-up failures).

Variables	N
Treatment failure	01
Follow up failure	33
Pregnancy during the study	02
Loss of medication or use of < 80% of the capsules	03
Did not complete final questionnaires	02
Did not collect the control exams	10
Missed rescheduled consultation without warning/ Loss of phone contact and/or address	16
Total	34

Table S3 -Evolution of quality of life before and after treatment using the QSF 36 questionnaire according to study groups

Group	Time	Mean±SD	Median	RTE	RTE 95%CI		p-value		
					Lower	Upper	Group	Time	Group*Time
**Functional capacity**									
Acyclovir+Melatonin	1	79±20	85	0.470	0.378	0.567	0.535	0.822	0.817
Acyclovir+Melatonin	2	77±26	85	0.467	0.373	0.566			
Melatonin	1	84±19	90	0.544	0.427	0.654			
Melatonin	2	76±23	85	0.438	0.333	0.552			
Acyclovir	1	81±20	90	0.516	0.382	0.648			
Acyclovir	2	87±17	95	0.598	0.463	0.715			
**Physical aspects**									
Acyclovir+Melatonin	1	159±105	100	0.463	0.376	0.554	0.235	0.122	0.125
Acyclovir+Melatonin	2	152±127	100	0.433	0.337	0.538			
Melatonin	1	234±145	300	0.597	0.466	0.711			
Melatonin	2	134±109	100	0.398	0.303	0.506			
Acyclovir	1	217±148	250	0.571	0.432	0.696			
Acyclovir	2	232±148	250	0.588	0.443	0.715			
**Pain**									
Acyclovir+Melatonin	1	49±29	42	0.423	0.326	0.529	0.118	0.604	0.769
Acyclovir+Melatonin	2	56±25	48	0.476	0.376	0.579			
Melatonin	1	57±30	52	0.489	0.373	0.606			
Melatonin	2	55±25	61	0.468	0.365	0.575			
Acyclovir	1	67±25	62	0.584	0.454	0.700			
Acyclovir	2	69±29	72	0.620	0.481	0.736			
**General State of Health**									
Acyclovir+Melatonin	1	57±21	57	0.420	0.319	0.532	0.809	0.153	0.328
Acyclovir+Melatonin	2	65±16	62	0.522	0.425	0.617			
Melatonin	1	63±23	67	0.521	0.396	0.643			
Melatonin	2	63±14	62	0.507	0.411	0.602			
Acyclovir	1	62±23	65	0.500	0.353	0.647			

Acyclovir	2	65±19	67	0.549	0.426	0.663			
**Vitality**									
Acyclovir+Melatonin	1	48±21	50	0.445	0.340	0.557	0.595	0.730	0.797
Acyclovir+Melatonin	2	50±19	50	0.464	0.372	0.561			
Melatonin	1	52±22	55	0.513	0.380	0.643			
Melatonin	2	55±14	55	0.547	0.442	0.645			
Acyclovir	1	54±16	55	0.538	0.419	0.652			
Acyclovir	2	54±15	50	0.521	0.403	0.635			
**Social aspects**									
Acyclovir+Melatonin	1	57±26	63	0.427	0.337	0.526	0.986	0.404	0.065
Acyclovir+Melatonin	2	70±23	63	0.567	0.471	0.655			
Melatonin	1	67±26	63	0.519	0.401	0.634			
Melatonin	2	64±22	63	0.496	0.396	0.597			
Acyclovir	1	64±32	63	0.511	0.370	0.651			
Acyclovir	2	62±27	63	0.478	0.350	0.611			
**Emotional aspects**									
Acyclovir+Melatonin	1	171±142	100	0.475	0.372	0,581	0.656	0.200	0.045
Acyclovir+Melatonin	2	158±127	100	0.470	0.378	0,566			
Melatonin	1	226±145	233	0.604	0.481	0,710			
Melatonin	2	125±123	100	0.394	0.293	0.509			
Acyclovir	1	191±155	100	0.520	0.383	0.653			
Acyclovir	2	207±144	200	0.565	0.436	0.683			
**Mental health**									
Acyclovir+Melatonin	1	54±21	54	0.480	0.374	0.588	0.817	0.733	0.405
Acyclovir+Melatonin	2	58±20	56	0.531	0.435	0.624			
Melatonin	1	57±20	56	0.533	0.417	0.644			
Melatonin	2	57±18	52	0.506	0.391	0.621			
Acyclovir	1	56±20	64	0.497	0.369	0.626			
Acyclovir	2	52±19	44	0.438	0.320	0.566			

p-values based on nonparametric repeated measures ANOVA model
